# Complete brachial plexus palsy following shoulder dislocation due to sneeze: a case report

**DOI:** 10.1186/s12245-019-0245-8

**Published:** 2019-09-13

**Authors:** Austin H. Middleton, John Roffers, Dean W. Ziegler

**Affiliations:** 1 Medical College of Wisconsin, 8701 W Watertown Plank Rd, Wauwatosa, WI 53226 USA; 2 Orthopedic Hospital of Wisconsin, 475 W River Woods Pkwy, Glendale, WI 53212 USA; 3 Blount Orthopaedic Associates, 525 W River Woods Pkwy Ste 100, Glendale, WI 53212 USA

**Keywords:** Brachial plexus, Dislocation, Sneeze, Brachial plexus injury

## Abstract

**Background:**

Traumatic shoulder dislocation is a frequent condition presenting to the emergency department. Due to the anatomy of the shoulder, associated neurovascular damage is not uncommon. Although clinical intuition may suggest that a higher-energy mechanism is required to produce neurovascular sequelae, the existing literature does not support this supposition.

**Case presentation:**

A 55-year-old woman presented to the emergency department with a complete brachial plexus palsy from an acute anterior shoulder dislocation following a violent sneeze. The shoulder was reduced without difficulty in the emergency department within 90 min of dislocation, and the patient was discharged. Her neurologic deficits gradually improved through a program of supervised therapy and orthopedic care. Follow-up at 1 year revealed marked improvement of motor and sensory function of the affected extremity with mild residual weakness and paresthesias in the affected hand.

**Conclusion:**

Neurovascular injuries in the setting of shoulder dislocation may be present despite low-energy injury mechanisms.

## Background

Traumatic anterior shoulder dislocation is the most common joint dislocation, affecting 44.3 people per 100,000 annually [1]. Individuals are at risk for neurologic and vascular complications following shoulder dislocation due to the proximity of the brachial plexus and the axillary artery. We present a case in which a patient sustained a complete brachial plexus palsy following anterior shoulder dislocation from a violent sneeze.

Written informed consent was obtained from the patient for publication of this case report.

## Case presentation

**Fig. 1:**
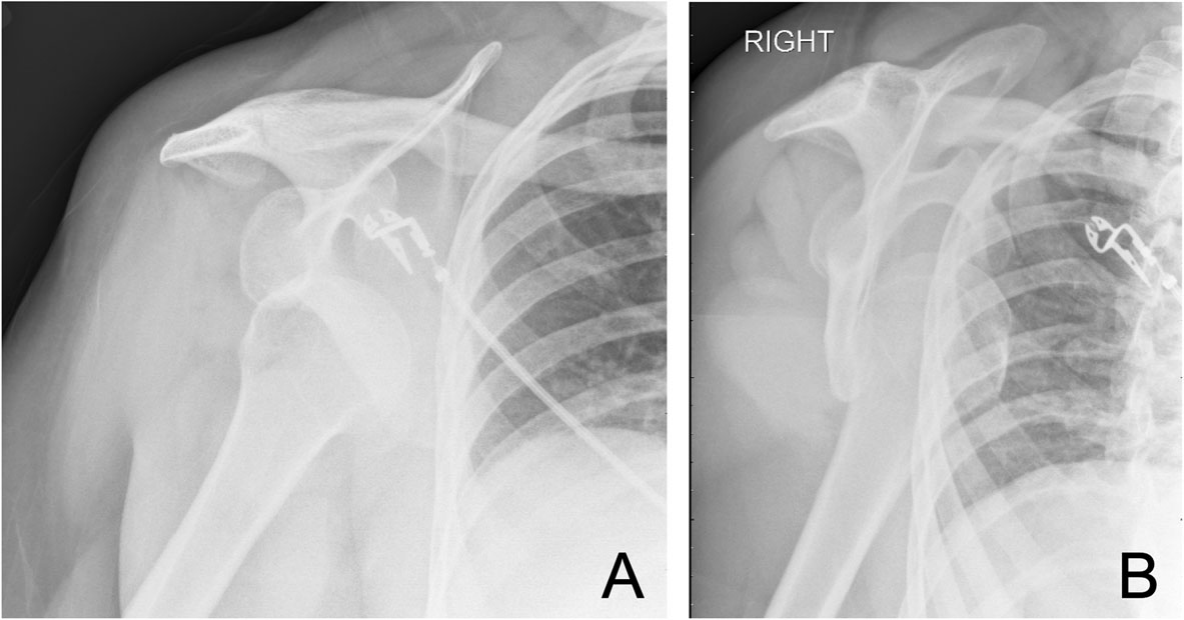
Pre-reduction plain radiographs. **a** AP view of the right shoulder prior to reduction. **b** Scapular Y view of the right shoulder prior to reduction

**Fig. 2:**
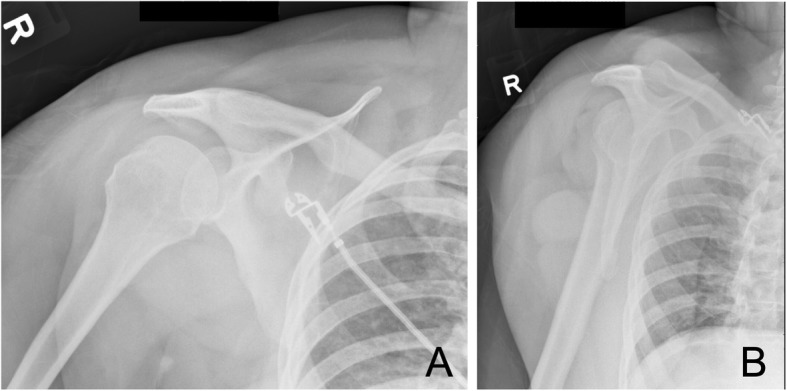
Post-reduction plain radiographs. **a** AP view of the right shoulder following reduction. **b** Scapular Y view of the right shoulder following reduction

A 55-year-old woman presented to a community hospital emergency department with an acute anterior right shoulder dislocation with ipsilateral upper extremity paresthesias and weakness following a violent sneeze. The patient’s past medical history included steroid-dependent asthma, type 2 diabetes, and class 1 obesity (BMI 34). Her surgical history was significant for an uncomplicated right shoulder arthroscopy and arthroscopic capsular release for adhesive capsulitis of the shoulder 10 years previously. A neurologic examination in the emergency department was performed prior to joint reduction and confirmed marked ipsilateral sensory and motor loss of the upper extremity. There was no evidence of any other cognitive or neurologic deficit. Plain radiographs were obtained, including AP and scapular Y views, demonstrating an anterior dislocation without evidence of acute fracture (Fig. 1). The shoulder dislocation was then uneventfully reduced within 90 min of dislocation with radiographic confirmation (Fig. 2). Computed tomography (CT) of the head was obtained due to the severity of her neurologic deficits. The CT findings were unremarkable. She was discharged from the emergency department in a shoulder immobilizer with instructions to follow up with her orthopedist. At 1-week follow-up with her orthopedic physician, the exam demonstrated persistent loss of motor and sensory function. Electromyography and nerve conduction studies (EMG/NCS) were obtained 2 weeks later and confirmed a severe multi-trunk brachial plexopathy characterized by both conduction block and motor axon loss. Magnetic resonance imaging (MRI) of the cervical spine was obtained to rule out cervical root avulsion but revealed only mild spondylosis. Over the next 6 weeks, the patient demonstrated dramatic motor and sensory improvement while participating in a program of physical and occupational therapy. One year after the original injury, her exam revealed only mild motor and sensory impairment in the affected hand.

## Discussion

Nerve injury is not uncommon following shoulder dislocation. Previous studies have reported the incidence between 12 and 13.5% [1, 2]. Despite the abundance of literature on neurological injury associated with shoulder dislocation, very few studies specifically report on the incidence of complete brachial plexus palsy. Robinson et al. found that single nerve lesions accounted for approximately 91% of neurological injuries [1]. A complete brachial plexopathy is therefore an uncommon complication of shoulder dislocation.

A number of risk factors have been implicated in the occurrence of nerve injury secondary to shoulder dislocation, including increased patient age [1–3], female sex [1], delayed reduction of the dislocation [2, 4], and coexisting rotator cuff tear or greater tuberosity fracture [1]. Counterintuitively, low-energy mechanisms of injury, such as falls, are more likely to produce neurologic injury than high-energy mechanisms [1]. Robinson et al. proposed that this finding may in fact be a surrogate marker for frailty, since their report demonstrated multi-nerve injuries were more common in patients over the age of 60 [1]. Further, Soldado et al. report that the most common mechanism of injury producing complete brachial plexus injury is a vertical impact to the shoulder, typically from an inferiorly directed force [5]. These associations may assist an emergency physician in recognition of severe neurologic injury in settings when intuition would not suggest that a sneeze could be responsible for such significant morbidity.

Complete brachial plexus palsy should be considered when a patient presents with profound weakness and sensory loss isolated to the affected limb. On physical exam, they may be unable to squeeze their hand (median/ulnar nerve), flex (median nerve) or extend (radial nerve) their wrist, flex their elbow (musculocutaneous nerve), or abduct their arm (axillary nerve). They will also demonstrate some level of asymmetric sensory impairment. These findings may help differentiate this type of injury from other peripheral neurological injuries (mono vs. polyneuropathy). While central nervous system pathology must be considered, it would be unusual to see combined motor and sensory findings isolated to a single limb, particularly in the setting of a shoulder dislocation.

In an acute setting of weakness in shoulder abduction, it may be difficult to distinguish rotator cuff tear (RCT) from neurologic injury. Careful examination of post-reduction radiographs should evaluate for the presence of greater tuberosity disruption, and MRI or computed tomography arthrography could be considered to confirm RCT [6]. It is also important to recognize the uncommon but devastating association vascular injury in patients with shoulder dislocation. Arterial injury is suggested in patients with delayed onset or progressive neuropathy or worsening pain following reduction. Urgent treatment is necessary in such cases to preserve viability of the limb and minimize long-term morbidity [7].

We manage acute anterior shoulder dislocations with the 0/90 program. This includes active/assisted external rotation to a goal/limit of 0° (neutral) and forward elevation in the frontal plane to a goal/limit of 90° for the first 3–4 weeks. Patients can immediately start external/internal rotator and deltoid isometrics at the side as well as active elbow range of motion and grip strength. A sling is also provided for comfort and stability when the patient is in an uncontrolled environment.

If neurological deficits persist beyond 3 weeks, electromyography may be helpful to define the extent of nerve injury [8, 9]. Imaging to evaluate for nerve root avulsion or lesions of the plexus may be indicated [6, 9]. Despite the initial presentation of a complete brachial plexus injury, substantial neurologic recovery has been demonstrated. Residual deficits of the intrinsic muscles of the hands are common, especially in those over the age of 50 [10].

## Conclusion

Although intuition may suggest that significant neurological injury would occur more commonly in the setting of high-energy trauma, this case demonstrates that substantial neurologic deficits may be associated with shoulder dislocation regardless of the mechanism of injury.

## Data Availability

Not applicable.

## Abbreviations

CT: Computed tomography
EMG/NCS: Electromyography and nerve conduction studies
MRI: Magnetic resonance imaging
RCT: Rotator cuff tear
